# Parasitic Mite Fauna in Asian Poultry Farming Systems

**DOI:** 10.3389/fvets.2020.00400

**Published:** 2020-07-09

**Authors:** Olivier A. E. Sparagano, Jeffery Ho

**Affiliations:** Department of Infectious Diseases and Public Health, Jockey Club College of Veterinary Medicine and Life Sciences, City University of Hong Kong, Kowloon, Hong Kong SAR, China

**Keywords:** *Dermanyssus gallinae*, *Ornithonyssus sylviarum*, *Ornithonyssus bursa*, ectoparasite, zoonosis, one health

## Abstract

The ubiquitous presence of hematophagous avian mites threatens the poultry industry in Asia and worldwide, adversely affecting the quality and quantity of eggs and poultry meat produced by affected flocks. This leads to considerable economic loss and welfare-related issues. The role of these blood-feeding arthropods as disease vectors is increasingly recognized as they may carry important zoonotic and epizootic pathogens. The poultry mites, *Dermanyssus gallinae* (Poultry Red Mite—PRM), *Ornithonysus sylviarum* (Northern Fowl Mite—NFM), and *Ornithonyssus bursa* (Tropical Fowl Mite—TFM) are endemic species across the Asian continent. In less frequency, scaly leg mite, depluming mite, and fowl cyst mite were documented. Considering global climate change and the popularity of backyard farming, the incidence of avian mite infestation is expected to increase as Asian production expands. The TFM may start to colonize sub-tropical nations where the seasonal temperature is comparable to tropical regions. Pyrethroids, organophosphates, carbamates, and macrocyclic lactones are licensed acaricides for use in China, Japan, and India. In recent years, the development of acaricide resistance has compromised the efficacy of chemical control measures. Several botanical acaricides based on plant and fungal constituents are being investigated. Judicious integration of multiple approaches to manage poultry mite infestation is advised. In this article, we review the prevalence, geographical distribution, zoonotic potential, and control measures of avian mites in poultry farms in Asia.

## Introduction

Mite infestations are of significant concern in the poultry farming industry, affecting the physical and psychological well-being of birds and the quality of egg production. Common avian mites include the poultry red mite (PRM), *Dermanyssus gallinae* (De Geer, 1778; Mesostigmata: Dermanyssidae), the northern fowl mite (NFM), *Ornithonyssus sylviarum* (Canestrini and Fanzago, 1877), and the tropical fowl mite (TFM), *Ornithonyssus bursa* (Mesostigmata: Macronyssidae; Berlese 1888). These mesostigmatic mites are obligatory hematophagous ectoparasites which feed on broilers and egg laying hens (1–3). They frequently colonize the vent feathers, which are warm and humid (3). Hens infested by these poultry mites produced fewer eggs with blood spots, rendering them unsellable (4–7). There are also poultry welfare concerns as heavy infestation could irritate the birds and even death of the flocks due to anemia (1–3). The ability of these mites to survive without attaching to the hosts for a considerable length of time render them difficult to be eliminated.

The rapid human population growth in Asia signifies an ever-increasing demand for food including poultry meat. To date, more than 60% of the world population is inhabited in the Asian continent. The past decades have witnessed a considerable shift of the global poultry market shares from North America and Europe to Asia, where it contributed more than 60% of the global poultry meat and egg production in the twenty-first century (8). Surveys into prevalence and distribution of poultry mites are mostly conducted in North America and Europe and are largely limited to PRM and NFM. To date, there is no review on Asian poultry mites.

In this review, literature search was conducted using title/abstract words including “poultry,” “mites,” and names of Asian countries without language restriction. Non-English articles were translated into English using Google-translate. The following search engines were used: Google Scholar, Scopus, and Web of Science. We discuss the epidemiology, characteristics and control measures of parasitic mites in Asian poultry farming systems. Other fowl ectoparasites such as lice and ticks are not covered in the current review.

## Prevalence and Geographic Distribution

Of 50 countries or regions in Asia as defined by the United Nations (9), studies on poultry mites were conducted in 10 countries; including Russia (10–12), China (13, 14), Japan (15–19), Malaysia (20), Myanmar (21), Iran (22–28), India (29, 30), Pakistan (31–34), Vietnam (35), and Saudi Arabia (36). The poultry mite fauna comprises four orders: mesostigmata, sarcoptiformes, acariformes, and trombidiformes.

### *Dermanyssus galliane* 

PRM is the most prevalent in Asia and worldwide (2, 3, 37). More than 46% of the farming systems in China, Japan, and Russia were infested by PRM (11, 12, 15). The PRM has variable morphology and genetic plasticity (38). These mites feed on hens occasionally at night and hide in crevices during daytime to avoid acaricide treatment, facilitating its persistence between flocks. Between 2008 and 2009, poultry farmers across 11 provinces in China were invited to participate in a large-scale cross-sectional study on prevalence of ectoparasites in commercial layer farms and broiler breeder farms. Fowl feathers and dust from cracks or crevices in poultry housing facilities were collected as specimens in Ziplock bags (12). More than 800 specimens were collected from farms collectively housing 5.5 million layer hens and 4.2 million of parent hens, representing more than 50% of the poultry production in China. Of 833 specimens, more than 80% were positive for at least one mite species or other ectoparasites. The PRM was present in 64% of commercial laying hens and 37% of breeder hens. Likewise, our recent review indicated that the prevalence of PRM was higher in layer hens (85.2%) than in broilers (0.6%) (1). In Iran, PRM was also highly prevalent in the majority of farms (22–24). Of note, inspection of eight caged layer farms and four breeder flock premises yielded PRM in all of the facilities (22). It should be noted that the latter study sampled from farms where farmers noticed ectoparasite infestation in their farms. This may explain the high prevalence in that study (22).

The prevalence rate of PRM in Asia was similar to that in Europe (1) and Africa (39–42). Although some studies reported a low prevalence of PRM and other mite species, it should be noted that these studies lacked random sampling and were largely limited to a few farms, which may not be representative of the countrywide situation. The dry and hot tropical climate in some Asian countries may render arthropods susceptible to dehydration and thus hindering their persistence in the environment. Temperature of the barns may not be well-controlled due to minimal resources available in some developing countries. For instance, the prevalence of PRM in North Africa and Iran, the prevalence of PRM varied across studies between 11% and up to 100% (23–25, 41, 42).

### *Ornithonyssus* spp.

The NFM and TFM are the important *Ornithonyssus* spp. responsible for poultry infestation. They are known to be prevalent in temperate and tropical regions, respectively. In Myanmar, four out of five premises were inhabited by either NFM or TFM (21). Interestingly, no PRM was isolated from any of the farms (21). In China, NFM was more frequently identified in breeders (46.9%) than in commercial layer hens (22.7%) (13). Another Chinese study was conducted in seven poultry housing premises across Hainan Island (China), where the climate ranged from tropical to subtropical (14). This study reported a prevalence rate of NFM as 42.8%, which was similar to another study conducted in Northern Chinese poultry farms (46.9%) (13). In the tropical Hyderabad region of India, TFM was frequently isolated from cage fittings, beneath feed troughs, fastening clips, under egg conveyer belts, and under manure belts across five breeding and caged layer poultry facilities (30). In Iran, TFM was found in more than 8% of the breeder flocks in the northern region warm and humid weather (23, 25). Likewise, in hot climate location in South America, such as Brazil, the TFM was the most commonly found mite species (*n* = 24,274) (43).

### *Knemidocoptes* and *Laminosioptes*

Sarcoptiforms comprise scaly leg mite (SLM), *Knemidocoptes mutans*, and depluming mite, *Knemidocoptes gallinae*. These are related mite species that burrow under the skin and lay eggs in the subcutaneous layer. Depluming mites mostly colonize ventral wings and the abdomen. The affected feathers became more susceptible to breakage. In addition, depluming mites burrow under the skin causing irritation to the birds. Most of the birds would try to pull out the affected feathers and thus creating lesions. In Russia, between 17.7 and 76% of the domestic fowls were infested by either SLM or depluming mite (12). In India, skin scraping of a male Aseel chicken with whitish film layer on legs and focal sloughing of the epidermis revealed the presence of larval and nymphal stages of the scaly leg mite.

The acarine *Laminosioptes cysticola* (Acariformes: Laminosioptidae, Vizioli, 1870), the fowl cyst mite (FCM), was reported in broiler farms in India (26). The research team monitored a group of 400 broilers over 3 years estimated 2.75% prevalence of FCM by microscopic examination. FCM formed cysts underneath the poultry skin as the female mites laid eggs. Superimposed bacterial infection frequently ensued (26). While the pathogenicity of FCM does not always lead to mortality, this mite species considerably distort the quality of meat in broilers rendering it unsellable to markets.

**Figure 1:**
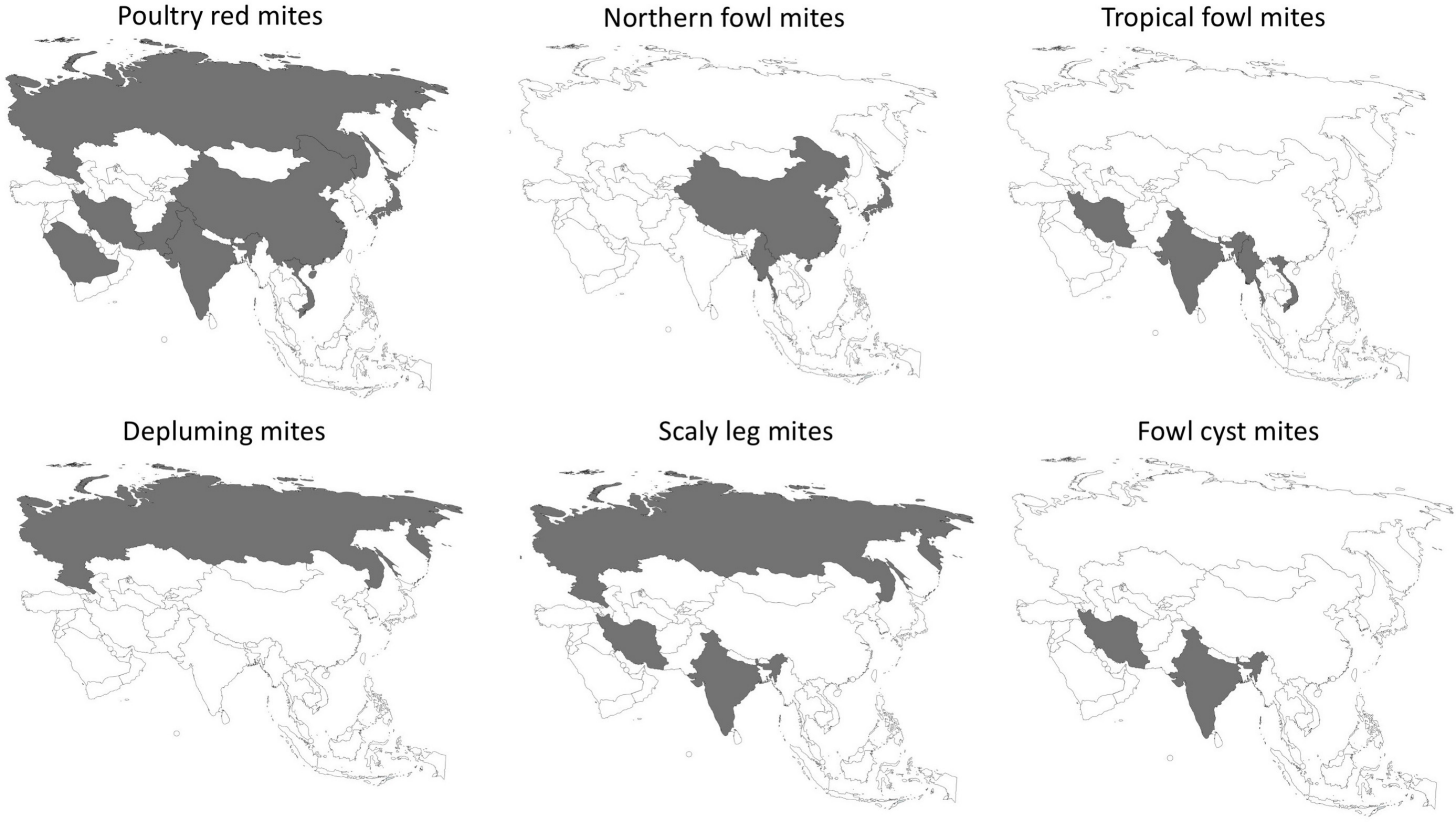
Geographical distribution of mite fauna in Asian poultry farming system (12–36).

The epidemiology of poultry mites across Asia are summarized in Table 1. Prevalence and distribution is depicted in Figure 1.

**Table 1:** Summary of studies with prevalence estimates on parasitic mite fauna in Asian poultry farming systems.

**Country (References)**	**Sample^a^ mean flock size or range**	**Mite**	**Prevalence (%) by production system**		
			**Cage**	**Backyard**	**Unknown**
Russia (12)	600	*D. gallinae*	-	55.7	-
		*K. mutans*	-	17.7	-
		*K. gallinae*	-	17.7	-
China (13)	6,360,200 layers	*D. gallinae*	64.1	-	-
		*O. sylviarum*	22.7	-	-
	5,534,300 breeders	*D. gallinae*	36.8	-	-
		*O. sylviarum*	46.9		-
China (14)	281,000	*O. sylviarum*	42.8	-	-
Japan (16)	700	*D. gallinae*	-	-	46.9
		*O. sylviarum*	-	-	17.3
Myanmar (21)	20 farms	*O. bursa*	-	-	10
		*O. sylviarum*	-	-	15
Iran (24)	600	*D. gallinae*	-	-	11
Iran (25)	600	*K. mutans*	-	7	-
		*D. gallinae*	-	26.3	-
		*O. bursa*	-	8.5	-
Iran (26)	400 broilers	*L. cysticola*	-	-	2.75
Pakistan (33)	Not reported	*D. gallinae*	14	-	-
		Laelaptidae	16	-	-
		Macrochelidae	57	-	-

a *Sample is presented in mean flock size or range unless otherwise stated by the authors*.

*The types of flocks are stated according to the description in references*.

## Co-Occurrence of Mite Infestation With Other Ectoparasites

Mite infestation in poultry frequently co-occur with other ectoparasites. It was reported that as much as 70% of commercial hens were parasitized at the same time by mites and other ectoparasites such as lice, fleas, and soft ticks (24, 25, 27). In Ethiopia, more than 34% of SLM were present in flocks from which fleas and lice were also isolated (40). Likewise, PRM was isolated from poultry farms together with ticks and lice (32). In contrast, in a Scottish smallholding where an adult cockerel and two young chicks were dead, the birds were found to be infested with PRM and depluming mite without other ectoparasites (44). Accordingly, we hypothesized that the ecological competition between mites and other ectoparasites such as lice, fleas and ticks may be more important in Asia than in Europe.

## Morphological and Molecular Characterization

Numerous studies employed microscopic morphology for the sole species identification method. Considering morphological similarity between different mite species and that morphological characteristics may be present shortly in a particular developmental stage, microscopic analysis alone may be limited. Differentiation between NFM and PRM requires specialized keys for identification of distinct characteristics in the dorsal shield, epigynal shields, and the chelicera (45). The advance of molecular technology in the last decade such as phylogenetic analysis provided evidence of epidemiological relatedness between mites. This relationship was not possibly determined by microscopic observation. The most commonly used polymorphic gene for this purpose is mitochondrial *cytochrome oxidase subunit 1* (COI) (14, 15, 46).

Applying COI gene sequencing could improve the accuracy of mite species prevalence estimation and reveal the possible transmission of mite species. For instance, in Japan, sequencing COI gene of 239 specimens led to identification of 28 haplotypes of PRM (15). Of these, two haplogroups were found identical to the European PRM clones. This information suggested possible importation of these mite haplogroups from Europe to Japan through international trade of infested chickens and poultry farm materials (15). In China, Bhowmick et al. (14) sequenced the COI marker gene to determine the prevalence of NFM. They found that all NFM isolates in Hainan were closely related. The majority of the haplotypes differed from each other by only single nucleotide (14).

## Potential Phoretic Association Between Poultry Mites, Wild Birds, and Flies

In many developing countries, the biosecurity level of poultry farms is low without proper hygiene. In Iran, for instance, more than 20% of the native fowls carried the poultry depluming mites (34). The commercial flocks were kept in free-range in a small-scale facility where they are at risk of contact with infested wild birds (34). Wild birds appeared to play a role in the dissemination of PRM among domestic birds in Brazil but not in Europe and Australia (47). It should be noted that the transmission of poultry mites between wild birds and commercial flocks remains elusive for other poultry mites. Further molecular characterization of mite fauna in commercial hens and proximal wild fowls in Asia will be required to confirm the hypothesis.

Diptera (true flies) are commonly found in poultry farms. Of 13,343 *Musca domestica* collected from poultry premises, 10 families of mites at different developmental stages were found. The most common one was *Macrocheles muscaedomesticae* (Mesostigmata: Macrochelidae), followed by Trombidiformes (Trombidiidae, Pygmephoridae, Tydeidae, Tarsonemidae, and Erythraeidae) and Sarcoptiformes (Pyroglyphidae, Histiostomatidae) (20). This study led to the hypothesis of insects as potential phoretic vectors for transmission of poultry mites.

## One Health-Related Issues

Beyond the poultry farming industry, the avian mites are of both veterinary and medical concerns due to their potential roles as vectors for bacterial and viral pathogens (48, 49) and possible host range expansion (50). Although the absolute vector competence of poultry mites has not yet been confirmed, the presence of epizootic and zoonotic pathogens in these mites is evident. The PRM is capable of carrying avipox virus, fowl adenovirus, Marek's disease virus, *Erysipelothrix rhusiopathiae, Salmonella enterica, Mycoplasma synoviae*, and *Mycoplasma gallisepticum* (17, 51). The proximity between poultry, domesticated birds, companion animals, and humans may facilitate alternate host adaptation of mesotigmatic mites (50). There is also controversy on whether different *Dermanyssus* subspecies infest chicken and pigeons (Personal communication). PRM has also been reported in cats and dogs (52, 53) and in humans as clinical dermatitis (also known as gamasoidosis) (54–56). Human cases of PRM infestations in Europe have been extensively reviewed (57). Other mesotigmatic mites including NFM and TFM have been isolated from people whose apartments were proximal to abandoned bird nests (54). In Asia, there were reports of human cases of NFM dermatitis in Japan (58) and PRM pruritus in Iran (55)

## Infestation Control Measures

### Chemical Methods

Traditionally, poultry mite management in livestock production relies on chemical acaricides. Chemical acaricides include organophosphates (e.g., Dichlorvos), pyrethroids (e.g., Cypermethrin, Deltamethrin), carbamates (Carbaryl), and macrocyclic lactones (Abamectin, Milbemectin). All of these were reported being used in mainland China (13) and Indian poultry farms (59). These groups of acaricides are also licensed for use in the veterinary market in Hong Kong (60) and Japan (18).

In mainland China, the majority of poultry farms (>50%) used either pyrethroids or organophosphates alone. More than 25% of poultry farmers do not re-treat their birds with acaricides within 2 weeks after the first treatment. This practice may lead to recolonization by the residual larva and promote chemical tolerance in mites. Although avermectins (i.e., abamectin and ivermectin) were not authorized for use in poultry flocks in mainland China, 63% farmers routinely supplement the bird feed with 1–2 ppm abamectin or ivermectin. This highlights the importance of education of farmers on appropriate use of acaricides and adherence to national guidelines.

Acaricide resistance in mites develop either as a result of increased metabolic breakdown of the acaricide or through acquisition of genetic mutation encoding an altered target with reduced affinity to the acaricide (61, 62). In Japan, 19.5% PRM in poultry farms showed resistance to three classes of acaricides, namely carbamates, pyrethroid, and organophoshates (18). Remarkably, the prevalence of PRM resistance to all commercially available acaricides increased from 13.7% in 2007–2010 to 18.3% in 2011–2013 (18). The occurrence of resistance against carbamate and combination insecticide [fenitrothion, permethrin, phthalthrin] appeared to be less frequent than those of other acaricides and insecticides (18). The underlying mechanism contributing to this phenomenon remains elusive.

To combat acaricide resistance in poultry mites, liquid preparation of diatomaceous earth, Silicon dioxide, was investigated. The insert dust is able to physically immobilize PRM and inhibits its locomotion. Coupling with mechanical cleaning, field trials confirmed that spraying 82% silica-containing Fisiocontrol (VetScience Bio Solutions) reduced up to 94% of the PRM population by 42 days after the first application (63).

### Biological Methods

Several plant extracts have been tested for use as botanical acaricides against poultry mites. In field trials, 10% garlic extract could considerably reduce mite infestation rate and restore erythrocyte and leucocyte counts in flocks (64, 65). Garlic extract is effective against NFM and PRM (28, 64). Some garlic-based products such as Garlic Barrier (Garlic Research Labs, United States) and Breck-a-Sol (ECOspray, United Kingdom) have already commercialized for controlling NFM and PRM (2). Laboratory *in vitro* studies on plant constituents revealed promising results of using plant-derived essential oils against PRM and NFM (66–69). Extracts from Samandua, Lychee, and Clove showed promising contact toxicity and vapor toxicity against PRM (67). Thyme and cade oils are effective against NFM (68).

Another biological control method is the use of entomopathogenic fungi such as *Aspergillus oryzae* and *Metarhizium* spp. against PRM (70, 71). Inoculation of *Metarhizium* strains at a concentration of 5 × 10^5^ condidia per cm^2^ reduced adult mite population by 56–95% in seven days (71). A comparable inoculum size of *A. oryzae* led to 10% higher mortality rate in treated PRM compared to the placebo group (70). Following field trials in poultry farms and appropriate control of dosage delivery, these fungal species may provide alternative options for biopesticides in the future.

Vaccination of poultry flocks against acarine has been increasingly recognized as a possible solution for arthropod control (2). Nonetheless, vaccine development is time-consuming and requires a thorough understanding of local epidemiology of poultry mites (2).

Recombinant akirin (Deg-AKR), calumenin (Deg-CALU), and *Rhipicephalus microplus* Subolesin (Rhm-SUB) have been identified as potential vaccine candidates (72, 73). Safety and technical issues have to be addressed before introducing arthropod vaccine into the poultry industry.

### Physical Method

The design of premises has long been recognized as an important determinant of ectoparasite infestation in poultry farms. The open systems such as free-range and backyard housing facilities are more prone to mite infestation as compared to the traditional caged system (1). Experimental studies and field trials suggested the possibility of using light regimen and gas for managing mite infestation. The population growth rate of PRM in rearing system under prolonged darkness (1: 23 h L:D) was three-fold higher than that with conventional lighting regimen (12: 12 h L:D) (74). Application of carbon dioxide could induce asphyxiation and thus reduce mite population by 85% within 24 h and 100% by 120 h (75).

Recently, the concept of integrated pest management has been extrapolated into use in poultry farming systems. The combination of chemical treatment, physical environmental control, and cultural interventions to control mite burden in poultry farming may reduce the risk of developing acaricides resistance and preserve the effectiveness of these armamentarium in the years to come. This kind of integrated mite infestation management program has been reviewed elsewhere (59).

## Concluding Remarks

Given the paucity of well-designed epidemiological studies on poultry arthropods in Asia, the prevalence of mite species circulating in farming systems remains elusive. The popularity of small-scale open farming systems in Asia may complicate the implementation of effective and affordable treatment strategies. Poultry mites frequently co-infest the same flocks with other ectoparasites. Further studies on the epidemiology of poultry mites and the interaction between mites and other ectoparasites are warranted to justify the use of appropriate control measures.

## Author Contributions

OS conceived the review topic, provided intellectual input, and critically revised the manuscript for submission. OS and JH performed literature searches. JH prepared the first draft of the manuscript and processed the data for the published figures. All authors contributed to the article and approved the submitted version.

## Conflict of Interest

The authors declare that the research was conducted in the absence of any commercial or financial relationships that could be construed as a potential conflict of interest.
